# Intake of mulberry 1-deoxynojirimycin prevents colorectal cancer in mice

**DOI:** 10.3164/jcbn.16-94

**Published:** 2017-05-16

**Authors:** Shuang E, Kazushi Yamamoto, Yu Sakamoto, Yui Mizowaki, Yui Iwagaki, Toshiyuki Kimura, Kiyotaka Nakagawa, Teruo Miyazawa, Tsuyoshi Tsuduki

**Affiliations:** 1 Laboratory of Food and Biomolecular Science, Graduate School of Agriculture, Tohoku University, 468-1 Aoba, Aramaki, Aoba-ku, Sendai 980-0845, Japan; 2 Institute of Food Research, National Agriculture and Food Research Organization (NARO), 2-1-12 Kannondai, Tsukuba, Ibaraki 305-8642, Japan; 3 Food and Biodynamic Chemistry Laboratory, Graduate School of Agriculture, Tohoku University, 468-1 Aoba, Aramaki, Aoba-ku, Sendai 980-0845, Japan

**Keywords:** azoxymethane, caloric restriction, colorectal cancer, 1-deoxynojirimycin, dextran sodium sulfate

## Abstract

The effect of 1-deoxynojirimycin, a caloric restriction mimetic, was examined in ICR mice with azoxymethane dextran sodium sulfate-induced colorectal cancer. Azoxymethane is a carcinogen (10 mg/kg body weight), and 2% dextran sodium sulfate (w/v) used as a colitis-inducing agent. Mice were separated into 5 groups: a group without colorectal cancer fed a normal diet (CO– group), and groups with colorectal cancer fed a normal diet (CO+ group), a calorie-restricted diet (caloric restriction group), and diets including 0.02% and 0.1% 1-deoxynojirimycin (l-1-deoxynojirimycin and H-1-deoxynojirimycin groups). The tumor incidence and number were reduced significantly in the caloric restriction group compared to the CO+ group, and were also suppressed in a dose-dependent manner by 1-deoxynojirimycin. mRNA for anti-apoptotic Bcl-2 was decreased and that for pro-apoptotic Bax was increased in the carcinoma tissue of CR, l-1-deoxynojirimycin and H-1-deoxynojirimycin groups. These results suggest that caloric restriction and 1-deoxynojirimycin inhibit growth of colorectal cancer by inducing apoptosis in an induced cancer model in mice.

## Introduction

Cancer is the leading cause of morbidity and mortality worldwide, with approximately 14 million new cases and 8.2 million cancer-related deaths in 2012.^(1)^ More than 60% of new cases annually occur in Africa, Asia and Central and South America, and among these, colorectal cancer is a major cause of tumor-related morbidity and mortality.^(1,2)^ This disease develops due to long-term exposure to environmental factors.^(3)^ In Japan, rapid Westernization of diet has increased the incidence and mortality of colorectal cancer, and this suggests that dietary treatment, especially caloric restriction (CR), may be effective for disease prevention. CR has beneficial effects on cancer prevention, with one study showing that the incidence of neoplasia with CR was significantly lower than that with an *ad libitum* diet.^(4,5)^ However, CR is accompanied by considerable stress in humans, which makes it difficult to use as a method for cancer prevention.

1-Deoxynojirimycin (DNJ) is a d-glucose analogue that is a characteristic constituent of mulberry (Moraceae) leaves. Dietary mulberry DNJ may be beneficial for suppression of abnormally high blood glucose.^(6)^ In addition, we have shown anti-obesity and anti-lipid peroxidation effects of DNJ, with decreased serum insulin and glucose, improved carbohydrate metabolism, and decreased lipid peroxide levels.^(7)^ These beneficial effects may occur because DNJ can reduce the bioavailability of glucose and have a CR effect. Moreover, DNJ intake showed changes in lipid metabolic parameters like CR.^(7)^ Cancer cells require larger amounts of glucose than normal cells, which suggests that growth of these cells might be inhibited by DNJ.^(8,9)^

Screening for agents for colorectal cancer prevention has been carried out in mouse models using the potent carcinogen azoxymethane (AOM), which induces colorectal cancers at a high incidence.^(10)^ Dextran sodium sulfate (DSS), a colitis-inducing agent, can be used after AOM to make a two-stage mouse colorectal cancer model.^(11,12)^ In this study, we used this two-stage model to examine the effect of DNJ on colorectal cancer. We also examined the mechanism of the DNJ effect by measuring the levels of apoptosis-related genes.

## Materials and Methods

### Materials

DNJ was extracted from mulberry leaves (Morus alba) and purified using ion-exchange chromatography followed by recrystallization.^(13)^ The purity of DNJ was shown to be >98% by hydrophilic interaction liquid chromatography with hybrid quadrupole/linear ion trap tandem mass spectrometry (HILIC-QTRAP MS/MS).^(7)^ NaCl, AOM, DSS, miglitol and 10% formalin were purchased from Wako Pure Chem. Ind. (Osaka, Japan).

### Animals and diets

All procedures were performed in accordance with the Animal Experiment Guidelines of Tohoku University. The animal protocol was approved by the Animal Use Committee at Tohoku University. Male ICR mice (*n* = 100, 4 weeks of age) and CE-2 (a control diet) were obtained from Japan Clea (Tokyo, Japan). Mice were housed with ten in each cage and with free access to the respective diets and distilled water in a temperature- and humidity-controlled room with light cycles of 12 h on and 12 h off.^(14)^ After being acclimatized to the control diet for one week, the 100 mice were randomly divided into 5 groups: control diet-fed mice without colorectal cancer inducement (CO–); and control (CO+), calorie-restricted (CR), low DNJ (L-DNJ) and high DNJ (H-DNJ) diet-fed mice with colorectal cancer inducement (*n* = 20 per group).

**Fig. 1:**
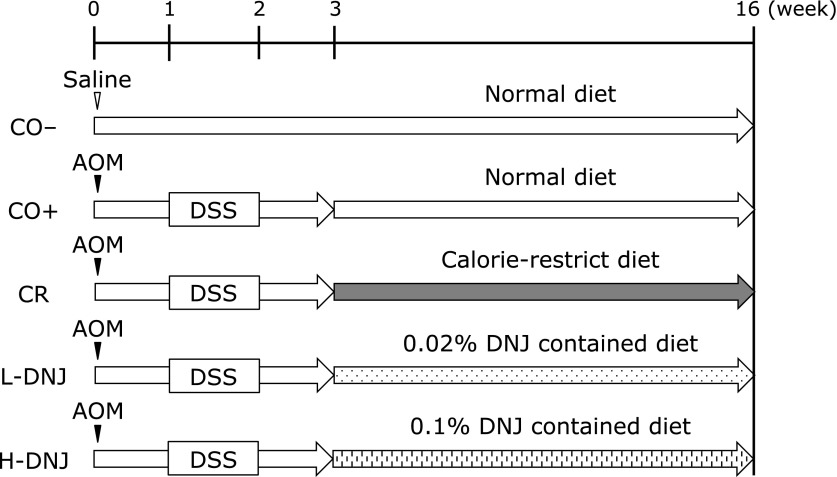
Study design. The CO− group received a single intraperitoneal injection of saline and a normal diet with no more treatment. The CO+, CR, L-DNJ and H-DNJ groups received a single intraperitoneal injection of AOM and 2% DSS in drinking water for 7 days, starting 1 week after the injection. From the third week, the groups received normal, caloric-restricted, 0.02% DNJ and 0.1% DNJ diets, respectively. After 16 weeks, all mice were sacrificed. AOM, azoxymethane; DNJ, 1-deoxynojirimycin; DSS, dextran sulfate sodium.

The experimental protocol is shown in Fig. 1. The CO+, CR, L-DNJ and H-DNJ groups received a single intraperitoneal injection of AOM in sterile saline at a dose of 10 mg/kg body weight to induce colorectal cancer. Starting one week after the injection, animals received 2% DSS in drinking water for one week to promote tumor progression. The CO– group received a single intraperitoneal injection of sterile saline only. The CO– and CO+ groups were fed CE-2 diet only. The CR, L-DNJ and H-DNJ groups were fed CE-2 diet for three weeks from the start of the experiment. Then, the CR group was fed every other day with CE-2 diet for 13 weeks, starting 1 week after cessation of DSS exposure. The L-DNJ and H-DNJ groups were fed CE-2 diet containing 0.02% and 0.1% DNJ, respectively, for 13 weeks, starting one week after cessation of DSS exposure. At the end of the 16-week period (21 weeks old), the mice were weighed and blood samples were collected after decapitation. Liver, kidney, pancreas, epididymis adipose tissue, and colon tissue were removed and weighed. The number of colorectal tumors detectable with the naked eye was measured. Serum was isolated by cold centrifugation at 1,000 × *g* for 15 min at 4°C (CAX-370 Hybrid Refrigerated Centrifuges, Tomy Digital Biology, Tokyo, Japan). Serum and tissue were stored at −80°C until use.

### Histological analysis of colon tissue

Colon tissue with or without tumors was fixed in 10% formalin and embedded in paraffin.^(15)^ Vertical sections (5 µm) were cut, mounted on glass slides, stained with hematoxylin and eosin (H&E), and observed using a microscope (BZ-9000; Keyence, Osaka, Japan).

### Biochemical analyses of serum and liver

To confirm a CR effect, biochemical analyses of serum and liver samples were performed as described previously.^(7,16)^ Triacylglycerol (TG) and total cholesterol (TC) levels in serum and liver, and phospholipid (PL), aspartate aminotransferase (AST), alanine aminotransferase (ALT), and glucose in serum were measured using commercial kits (Wako Pure Chem.). The PL content in liver was determined using the method described by Rouser (1970).^(17)^ Insulin levels in serum were determined using an ELISA kit (Shibayagi, Shibukawa, Japan).

### mRNA expression analyses

For real-time quantitative reverse transcriptase PCR (qRT-PCR), total RNA was isolated from colon (tumor and normal tissues) using a RNeasy Mini Kit (Qiagen, Valencia, CA), after elution with 30 µl of RNase-free water, and stored at −80°C until use.^(18)^ To quantify expression of genes, the mRNA levels of β-actin, B-cell lymphoma 2 (Bcl-2), Bcl-2 associated X protein (Bax) and sirtuin 1 (Sirt1) were determined using a TP870 Thermal Cycler Dice Real Time System (Takara Bio, Otsu, Japan). This system allows real-time quantitative detection of PCR products by measuring the increase in fluorescence caused by binding of SYBR green to double-stranded DNA. cDNA was synthesized from the total RNA in colon using a Ready-To-Go T-Primed First-Strand Kit (GE Healthcare, UK). The cDNA was subjected to PCR amplification using SYBR^®^ Premix Ex Taq^TM^ (Perfect Real Time, Takara Bio) and gene-specific primers for β-actin, Bcl-2, BAX or Sirt1. Primer sequences were as follows: β-actin (forward) 5'-AGT GTG ACG TTG ACA TCC GTA-3', β-actin (reverse) 5'-GCC AGA GCA GTA ATC TCC TTC T-3', Bcl-2 (forward) 5'-TGT GGT CCA TCT GAC CCT CC-3', Bcl-2 (reverse) 5'-ACA TCT CCC TGT TGA CGC TCT-3', BAX (forward) 5'-TGA AGA CAG GGG CCT TTT TG-3', BAX (reverse) 5'-AAT TCG CCG GAG ACA CTC G-3', Sirt1 (forward) 5'-GAC GAT GAC AGA ACG TCA CAC-3', Sirt1 (reverse) 5'-CGA GGA TCG GTG CCA ATC A-3'. The PCR conditions were 95°C for 10 s, and then 95°C for 5 s and 60°C for 31 s over 40 cycles for each gene. Melting curve analysis was performed following each reaction to confirm the presence of a single reaction product. The cycle threshold (CT) represents the PCR cycle at which the reporter fluorescence increased above a baseline signal. The ratio between the β-actin content in standard and test samples was defined as the normalization factor.

### Determination of lipid peroxides

To examine the reduction of oxidative stress caused by CR, the levels of thiobarbituric acid reactive substances (TBARS) in serum and liver were determined.^(7,14)^ To examine oxidative stress caused by AOM and DSS in colon tissue, TBARS level in normal colon tissue was determined.^(19)^

### DNJ concentration in colorectal cancer and normal tissue

DNJ concentrations in colon tissue (tumor or normal) were determined using HILIC MS/MS.^(17)^ In brief, a 1:10 dilution of colorectal cancer or normal tissue homogenate (500 µl), 100 µl of 0.1 µg/ml miglitol (internal standard) and 600 µl of acetonitrile were mixed by sonicating for 1 min and vortexing for 30 s. After centrifugation at 8,000 × *g* for 10 min at 4°C (CAX-370), the supernatant was collected. A 5-µl aliquot of the resulting extract was subjected to HILIC-MS/MS using a Shimadzu liquid chromatograph and a 4500 tandem mass spectrometer (AB Sciex, Tokyo, Japan). Under positive ion electrospray ionization conditions, MS/MS parameters were optimized with DNJ and miglitol. Samples (5 µl each) were separated on a HILIC column (TSK gel Amide-80, 4.6 mm × 150 mm; Tosoh, Tokyo, Japan), eluted with a mixture of acetonitrile and water (675:325, v/v) containing 6.5 mM ammonium formate (pH 5.5) at a flow rate of 0.2 ml/min and a temperature of 40°C. Post-column, DNJ was detected by HILIC-MS/MS with multiple reaction monitoring for transition of the parent ion to the product ion. DNJ concentrations were calculated using a calibration curve.

### Statistical analysis

Results are expressed as the mean ± SEM. Data were analyzed using a one-way ANOVA with a Tukey-Kramer test for multiple comparisons among three or four groups. A difference was considered to be significant at *p*<0.05.

## Results

### Effects of caloric restriction and DNJ on growth parameters

There were significant decreases in food and energy intake in the CR group compared to the CO− and CO+ groups. The CR group had caloric restriction of about 20% compared to the CO+ group. In contrast, there were significant increases in food and energy intake in the H-DNJ group compared to the CO− group (Table 1). There were no significant differences in body weight and tissue weights among the groups with induced colorectal cancer.

### Effects of caloric restriction and DNJ on tumor formation

**Fig. 2:**
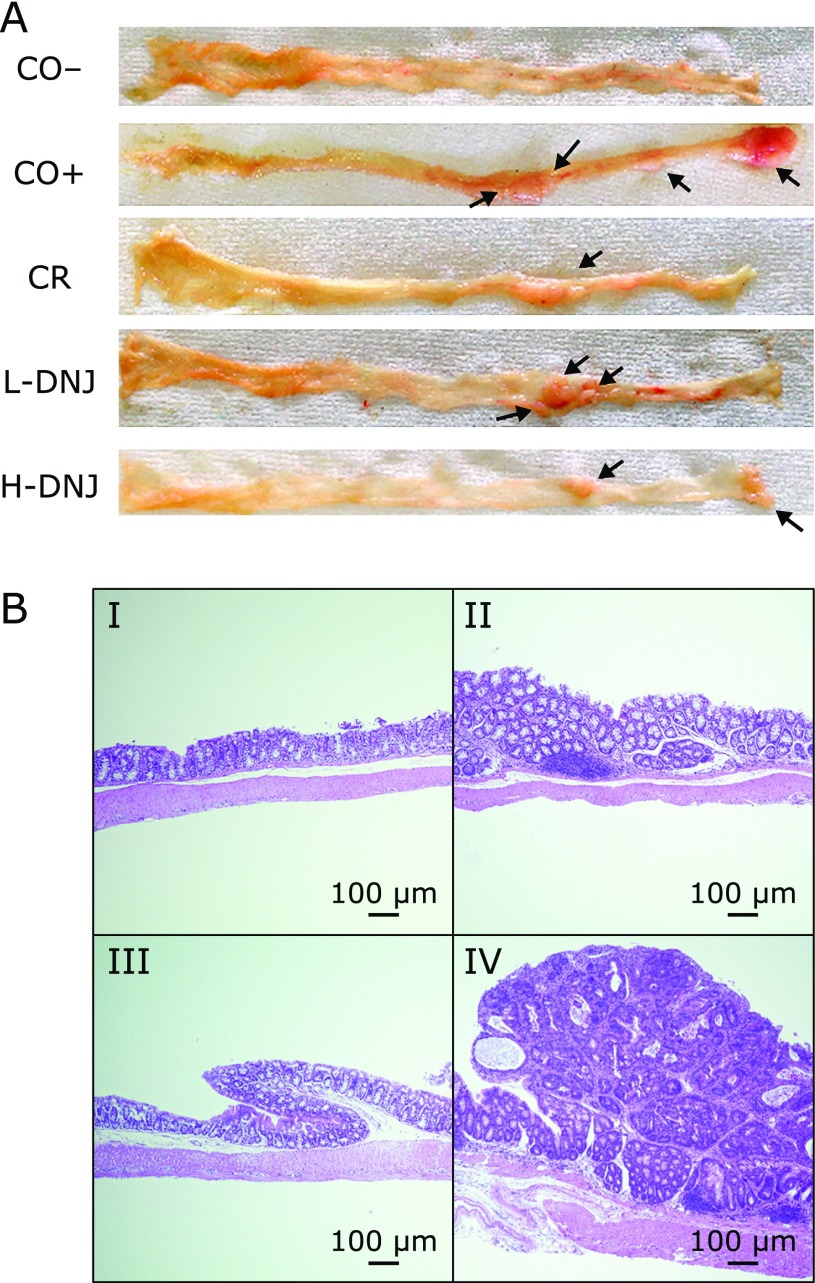
Effect of caloric restriction and DNJ on colon tissue in male mice with induced colorectal cancer. (A) Colon tissue in each group. Arrows indicate tumors. (B) Representative histology images from hematoxylin and eosin-stained colon specimens (magnification ×4): (I) normal colon, (II) colon tissue with mild pathology, (III) dysplastic crypts, and (IV) well-differentiated tubular adenocarcinoma. Bars indicate 100 µm.

**Fig. 3:**
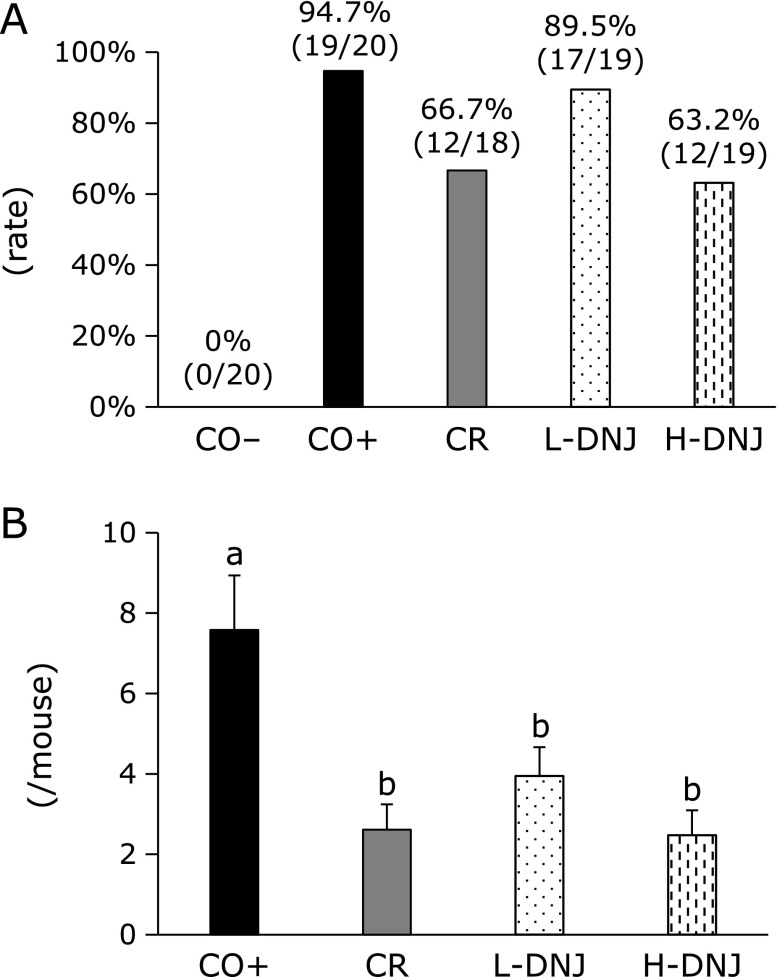
Effect of caloric restriction and DNJ on tumor incidence (A) and number (B) in male mice with induced colorectal cancer. Values are means ± SE, *n* = 18–20. ^a^^,^^b^*p*<0.05. DNJ, 1-deoxynojirimycin.

The number of colonic tumors visible with the naked eye was counted after sacrifice (Fig. 2A). H&E staining of colon tissues to confirm the occurrence of a tumor (adenoma and adenocarcinoma) showed colon inflammation (Fig. 2B-II), adenoma (Fig. 2B-III) and adenocarcinoma (Fig. 2B-IV) in mice with induced cancer, but normal tissue (Fig. 2B-I) in the control group (CO−). Tumor incidences and numbers are shown in Fig. 3. Compared to the CO+ group, there was a 28% decrease in tumor incidence in the CR group (Fig. 3A), and 5.2% and 31.5% decreases in the L-DNJ and H-DNJ groups, respectively. There were also significant decreases in the number of tumors in the CR, L-DNJ, and H-DNJ groups compared to the CO+ group (Fig. 3B). The incidence and number decreased in a DNJ dose-dependent manner, and the results in the H-DNJ group were similar to those in the CR group.

### Effects of caloric restriction and DNJ on serum and liver parameters

To investigate the effects of caloric restriction and DNJ on lipid and carbohydrate metabolism, serum levels of TG, TC, PL, glucose and insulin were determined (Table 2). There were significant increases in TC and PL in the L-DNJ group compared to the CO+, CR and H-DNJ groups. There was a significant decrease in insulin in the CR, L-DNJ and H-DNJ groups compared to the CO+ group. To evaluate the safety of caloric restriction and DNJ, serum and liver TBARS and serum ALT and AST levels were measured. There were significant decreases in serum and liver TBARS in the CR and H-DNJ groups compared to the CO− and CO+ groups, and TBARS decreased dose-dependently with DNJ. There were significant decreases in serum AST in the CR and H-DNJ group compared to the CO+ group. To investigate the effects of caloric restriction and DNJ on lipid metabolism, liver levels of TG, TC, and PL were determined (Table 2). There were no significant differences in liver parameters among the groups with induced colorectal cancer. There were no significant differences in serum and liver parameters between the CO− and CO+ groups. To investigate the effects of caloric restriction and DNJ on the oxidative stress caused by AOM and DSS in normal colon tissue, TBARS levels in the colon were determined (Table 2). There was significant increase in colon TBARS in the CO+ group compared to the CO− group. There were significant decreases in colon TBARS in CR, L-DNJ and H-DNJ groups compared to the CO+ group, and TBARS decreased dose-dependently with DNJ.

### Effects of caloric restriction and DNJ on apoptosis

**Fig. 4:**
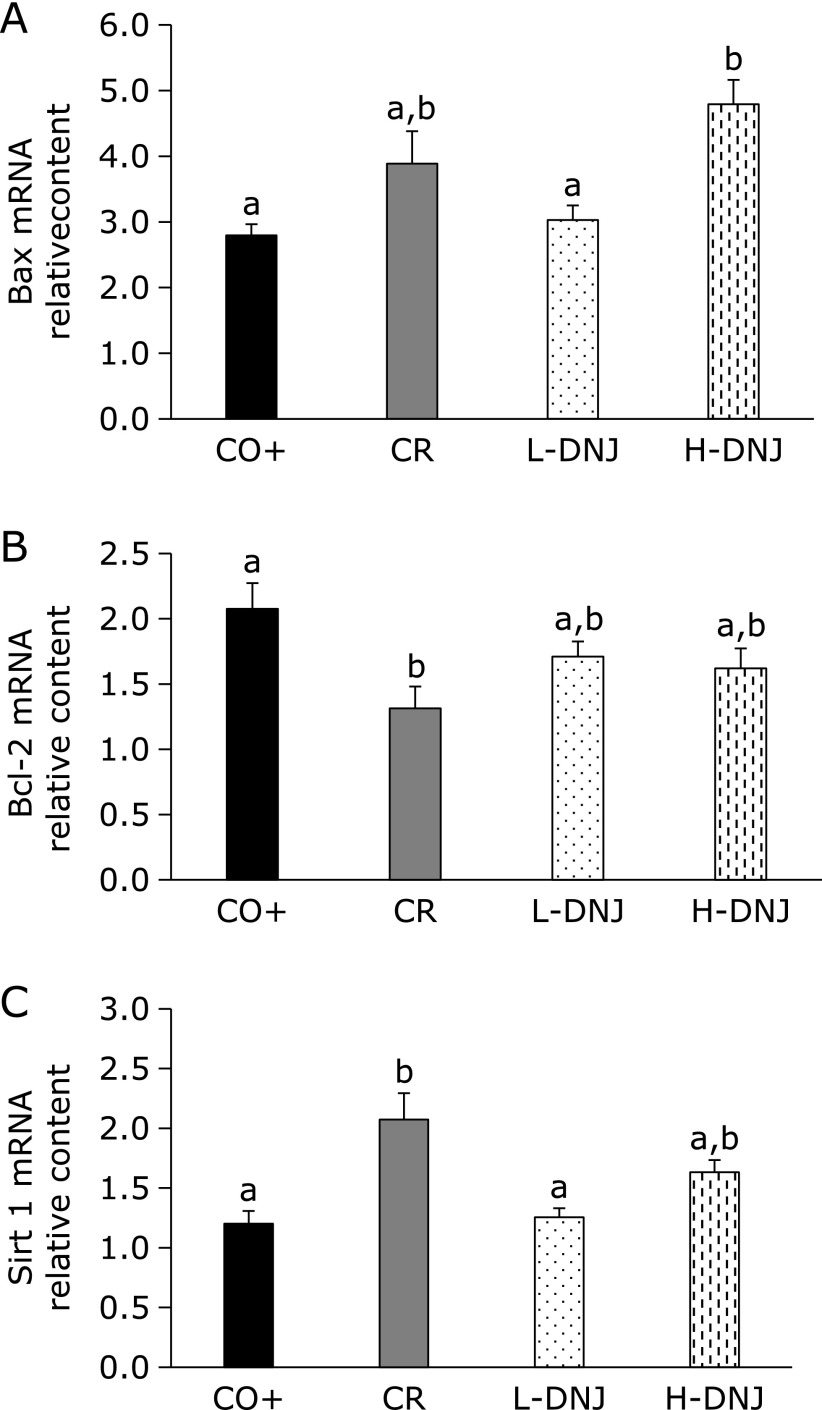
Effect of caloric restriction and DNJ on BAX (A), Bcl-2 (B), and Sirt 1 (C) mRNA levels in male mice with induced colorectal cancer. Values are means ± SE, *n* = 18–20. ^a^^,^^b^*p*<0.05. Bax, Bcl-2 associated X protein; Bcl-2, B-cell lymphoma 2; DNJ, 1-deoxynojirimycin; Sirt 1, sirtuin 1.

 To examine the tumor suppression mechanism, the mRNA levels of two apoptosis-related genes (Bcl-2 and BAX) were measured. There were significant increases in mRNA for pro-apoptotic Bax in the CR and H-DNJ groups compared to the CO+ and L-DNJ groups (Fig. 4A), and significant decreases in mRNA for the anti-apoptotic gene Bal-2 in the CR group compared to the CO+ group, with a tendency for decreases in the L-DNJ and H-DNJ groups (Fig. 4B). The mRNA levels for the two genes varied dose-dependently with DNJ. These findings suggest that caloric restriction and DNJ induce apoptosis of cancer cells. In addition, the caloric restriction-related gene Sirt1 was examined as a caloric restriction marker (Fig. 4C). There were significant increases in mRNA for Sirt1 in the CR group compared to the CO+ group, and tendencies for increases in the L-DNJ and H-DNJ groups.

### DNJ concentration in colorectal cancer and normal tissues

To determine whether DNJ is absorbed in colon tissue, DNJ was measured in normal and tumor colon tissues. DNJ was not found in mice without DNJ intake. The DNJ concentration in the H-DNJ group was significantly higher than that in the L-DNJ group in normal tissue (0.448 ± 0.114 vs 0.089 ± 0.013 ng/g) and tumor tissue (1.03 ± 0.09 vs 0.204 ± 0.048 ng/g). The DNJ concentration in tumor tissue was significantly higher than that in normal tissue.

## Discussion

In this study, we showed that caloric restriction inhibits AOM/DSS-induced colorectal cancer in ICR mice, and that DNJ suppresses this disease through a caloric restriction-like mechanism. This is the first report of a colorectal cancer preventive effect of DNJ.

Cancer cells require more energy, and especially more glucose, for growth compared to normal cells. This is referred to as the Warburg effect.^(8,20)^ Therefore, we speculated that caloric restriction can inhibit the growth of tumor cells. In this study, the growth of mice was not particularly affected by caloric restriction, since there was only 20% restriction in the CR group, but colorectal tumors were significantly reduced in the CR group compared to the CO+ group. This suggests that caloric restriction can have an inhibitory effect on colorectal cancer. Similarly, Derry *et al.*^(10)^ reported that 30% caloric restriction reduced the growth of colorectal cancer significantly. Similar results were also found by Olivo-Marston *et al.*^(4)^ and Reddy *et al.*^(15)^ The suppressive effect of DNJ on colorectal cancer occurred in a dose-dependent manner, and DNJ had no significant effect on mouse growth.

Sirt1 is involved in acute and chronic energy limitation, such as fasting and diet restriction, and controls metabolism by deactivating many transcriptional regulatory factors and affecting gene expression.^(21,22)^ Therefore, we used Sirt1 as a marker to judge the effect of caloric restriction on colorectal cancer tissue. Sirt1 was increased in the CR group and also increased in a DNJ dose-dependent manner. In addition, serum and liver parameters were measured to confirm a CR effect. Since the trend similar to the previous report was confirmed, it was objectively shown that DNJ has a CR effect.^(7)^ Thus, caloric restriction appears to be involved in one of the tumor suppressor mechanisms of DNJ.

Tumor suppression through caloric restriction occurs through induction of apoptosis in cancer cells.^(23)^ To confirm this mechanisms, we measured mRNA levels of the anti-apoptotic gene Bcl-2 and pro-apoptotic gene Bax.^(24–26)^ In caloric restriction, mRNA for Bax increased and mRNA for Bcl-2 decreased in cancer cells. Similar results were obtained with DNJ intake, which suggests that DNJ induces apoptosis in cancer cells through the Bcl-2/Bax signaling pathway. These findings are also consistent with the role of DNJ as a caloric restriction mimetic.

Absorption of DNJ was verified in normal and tumor colon tissues, which indicates that DNJ can act directly on cancer cells. In addition, the DNJ concentration in colorectal cancer tissue was higher than that in normal colon tissue in both DNJ groups. The similarity of the structure of DNJ to that of glucose may allow DNJ to be taken into cancer cells because these cells have a high demand for glucose compared to normal cells.^(8,20)^ Thus, DNJ may have greater effects on cancer cells than on normal cells.

As found previously, the levels of TBARS, an oxidative stress indicator, in serum and liver were reduced by DNJ intake.^(7)^ Moreover, in this study, TBARS level in colon was also reduced by DNJ intake. Oxidative stress promotes cancer, and thus DNJ may inhibit cancer growth by reducing oxidative stress.^(27,28)^ In addition, oxidative stress greatly affects the immune system such as promotion of inflammation.^(11,12)^ And, the immune system is closely related to the onset of colorectal cancer.^(29,30)^ Therefore, DNJ may inhibit cancer growth by affecting the immune system.

Caloric restriction is a potential approach to prevention of colorectal cancer, but eliminating food intake is also stressful. Therefore, a caloric restriction mimetic such as DNJ would be ideal for cancer prevention. In this study, we showed the efficacy of DNJ for this purpose. Determination of the appropriate dose of DNJ and understanding of the detailed mechanism of the colorectal cancer preventive effect of DNJ will require further studies.

## Figures and Tables

**Table 1:** Effect of calorie restriction and DNJ on growth parameters in colon cancer-induced male mice

	CO−	CO+	CR	L-DNJ	H-DNJ
Food intake (g/day)	5.41 ± 0.04^b^	5.69 ± 0.08^b^^,^^c^	4.45 ± 0.07^a^	5.66 ± 0.09^b^^,^^c^	5.72 ± 0.08^c^
Energy intake (kcal/day)	18.7 ± 0.15^b^	19.6 ± 0.28^b^^,^^c^	15.4 ± 0.20^a^	19.5 ± 0.31^b^^,^^c^	19.7 ± 0.27^c^
Final body weight (g)	44.5 ± 0.91	43.8 ± 0.79	41.4 ± 0.32	43.0 ± 0.39	41.7 ± 0.67
Tissue weight (g/100 g body weight)					
Liver	4.09 ± 0.06	4.49 ± 0.13	4.33 ± 0.15	4.47 ± 0.09	4.38 ± 0.13
Pancreas	0.85 ± 0.03	0.93 ± 0.04	0.90 ± 0.04	0.93 ± 0.04	0.92 ± 0.04
Kidney	1.82 ± 0.03^a^	1.92 ± 0.05^a^^,^^b^	1.90 ± 0.04^a^^,^^b^	2.03 ± 0.04^b^	1.89 ± 0.06^a^^,^^b^
Epididymis adipose	1.46 ± 0.18^a^	1.09 ± 0.12^a^^,^^b^	0.79 ± 0.12^b^	0.80 ± 0.11^b^	0.77 ± 0.13^b^

Values are means ± SE, *n* = 18–20. Means in a row with different letters are significantly different at *p*<0.05.

**Table 2:** Effect of calorie restriction and DNJ on plasma, liver and colon parameters in colon cancer-induced male mice

	CO−	CO+	CR	L-DNJ	H-DNJ
Serum					
Triacylglycerol (mmol/L)	1.51 ± 0.09	1.57 ± 0.09	1.50 ± 0.10	1.72 ± 0.14	1.45 ± 0.11
Total cholesterol (mmol/L)	2.32 ± 0.06^a^^,^^b^	2.19 ± 0.04^a^	2.11 ± 0.06^a^	2.55 ± 0.08^b^	2.23 ± 0.07^a^
Phospholipid (mmol/L)	2.24 ± 0.04^a^	2.15 ± 0.05^a^	2.14 ± 0.07^a^	2.51 ± 0.09^b^	2.24 ± 0.08^a^
Glucose (mmol/L)	4.57 ± 0.20	5.03 ± 0.26	5.00 ± 0.18	4.69 ± 0.21	5.04 ± 0.28
Insulin (mg/L)	0.24 ± 0.03^a^^,^^b^	0.26 ± 0.04^a^	0.15 ± 0.03^b^	0.15 ± 0.01^b^	0.15 ± 0.01^b^
HOMA-IR	1.00 ± 0.15	1.20 ± 0.20	0.66 ± 0.06	0.65 ± 0.06	0.66 ± 0.07
TBARS (µmol/L)	5.14 ± 0.21^a^	5.47 ± 0.48^a^	4.17 ± 0.23^b^	4.90 ± 0.29^a^^,^^b^	4.27 ± 0.25^b^
ALT (UI/L)	9.75 ± 0.42	10.4 ± 0.52	10.0 ± 0.54	9.50 ± 0.92	11.5 ± 0.57
AST (UI/L)	53.0 ± 5.44^a^	61.0 ± 7.89^b^	46.5 ± 5.28^a^	44.7 ± 3.32^a^	43.5 ± 2.41^a^
Liver					
Triacylglycerol (µmol/g)	12.2 ± 1.13	8.82 ± 0.84	11.0 ± 1.21	13.0 ± 1.34	10.7 ± 1.39
Total cholesterol (µmol/g)	7.68 ± 0.65	7.84 ± 0.73	7.02 ± 0.20	6.45 ± 0.50	6.55 ± 0.41
Phospholipid (µmol/g)	34.5 ± 0.8	32.7 ± 1.4	35.4 ± 1.0	35.5 ± 1.2	34.6 ± 1.4
TBARS (nmol/g)	68.9 ± 5.3^a^	63.6 ± 3.9^a^	44.7 ± 2.8^b^	59.5 ± 3.1^a^	41.1 ± 3.3^b^
Colon					
TBARS (nmol/g)	8.6 ± 0.7^a^	21.2 ± 1.3^c^	14.9 ± 0.9^b^	18.8 ± 1.0^c^	13.7 ± 1.1^b^

Values are means ± SE, *n* = 18–20. Means in a row with different letters are significantly different at *p*<0.05. HOMA-IR, homeostasis model assessment-insulin resistance; TBARS, thiobarbituric acid reactive substances; ALT, alanine transaminase; AST, aspartate aminotransferase.
